# Reactive oxygen species modulator 1 expression predicts lymph node metastasis and survival in early-stage non-small cell lung cancer

**DOI:** 10.1371/journal.pone.0239670

**Published:** 2020-12-01

**Authors:** Taehee Kim, Yoon Jin Cha, Ji Hyun Park, Arum Kim, Yong Jun Choi, Hye Jung Park

**Affiliations:** 1 Department of Internal Medicine, Kangnam Sacred Heart Hospital, Hallym University College of Medicine, Seoul, South Korea; 2 Department of Pathology, Gangnam Severance Hospital, Yonsei University College of Medicine, Seoul, South Korea; 3 Department of Internal Medicine, Gangnam Severance Hospital, Yonsei University College of Medicine, Seoul, South Korea; Seoul National University College of Pharmacy, REPUBLIC OF KOREA

## Abstract

Reactive oxygen species modulator 1 (romo1) causes cell hyperplasia and promotes cancer cell invasion. Based recent studies, the overexpression of romo1 is associated with lymphatic metastasis and poor prognosis in lung cancer. We aimed to evaluate associations between romo1 expression and lymph node metastasis in non-small cell lung cancer (NSCLC). Clinical data and pathological results were retrospectively reviewed for 98 subjects diagnosed with NSCLC and who underwent surgical biopsy between 1994 and 2009. A total 98 tumor specimens were analyzed. The romo1 H score was correlated with stage and was significantly higher in subjects with lymph node metastasis than in those without metastasis (173 vs 116; *P* < 0.05). The area (%) of grade 1 expression was significantly smaller (19.5 vs 37.0; *P* = 0.005) and the area of grade 3 expression was significantly larger (27.9 vs 6.00; *P* < 0.001) in subjects with lymph node metastasis than in those without metastasis. In stage I patients, disease free survival (DFS) (191 ± 18.8 vs. 75.6 ± 22.4 months, *P* = 0.004) was significantly longer in the low romo1 group than in the high romo1 group. A multivariate analysis showed a significant association between high romo1 expression and poor DFS (hazard ratio 5.59, 95 confidence interval, 1.54–20.3, *P* = 0.009). These findings support the prognostic value of romo1 in NSCLC, especially in stage I.

## Introduction

Reactive oxygen species modulator 1 (romo1) is a non-selective cation channel present on the surface of mitochondria and it produces reactive oxygen species (ROS) by oxidative stress [1, 2]. Increased ROS production by romo1 alters intracellular oxidative stress homeostasis, thereby inducing DNA damage and genomic instability [1]. Antioxidant defense mechanisms minimize toxicity induced by ROS. However, ROS production often exceeds the antioxidant capacity, leading to cell death, inflammation, or cancer cell production [3]. The overexpression of romo1 is frequently observed in various cancer cell lines. Increased ROS production induced by romo1 overexpression can cause persistent oxidative stress, increase malignancy, and promote cancer development and progression [4].

Recent studies have shown that romo1 is a diagnostic and prognostic factor in various cancers, including hepatocellular carcinoma and colorectal cancer [5, 6]. In lung cancer, oxidative stress is particularly important. Cigarette smoking is a strong causative agent for lung cancer mediated by oxidative stress [7]. Various mechanisms may explain the relationship between oxidative stress and lung cancer, including superoxide dismutases, glutathione peroxidases, heme oxygenases, and the NF-κB signaling pathway [8]. Although recent retrospective clinical studies have suggested that the overexpression of romo1 can be a diagnostic and prognostic marker for non-small cell lung cancer (NSCLC) [2, 9, 10], conclusive evidence is lacking.

Therefore, the aim of the present study was to investigate the clinical relevance of romo1 expression in patients with early stage NSCLC.

## Material and methods

### Study subjects and specimens

Tumor tissues were collected from patients with lung cancer who underwent surgical biopsy between November 1994 and July 2009 at Gangnam Severance Hospital. During this period, 134 surgical specimens were obtained from patients who received surgical biopsy; only 98 patients were diagnosed with NSCLC and had available electronic medical records. Computed tomography of the chest, magnetic resonance imaging of the brain and 18F-fluorodeoxyglucose positron-emission tomography for clinical staging were performed for all patients. Pathologic staging was determined according to the International Association for the Study of Lung Cancer TNM staging classification of NSCLC [11].

Clinical data up to December 31, 2018 were collected retrospectively by reviewing medical records. This retrospective study was approved by the Institutional Review Board of Yonsei University Gangnam Severance Hospital (3-2018-0130) and informed consent was waived. The research assistants collected all the required data including medical records and tissue samples, concealed any identifiable details about the patients. All the methods in the study were carried out with relevant guidelines and regulations according to the IRB recommendation.

### Immunohistochemical staining and romo1 scoring

**Fig 1:**
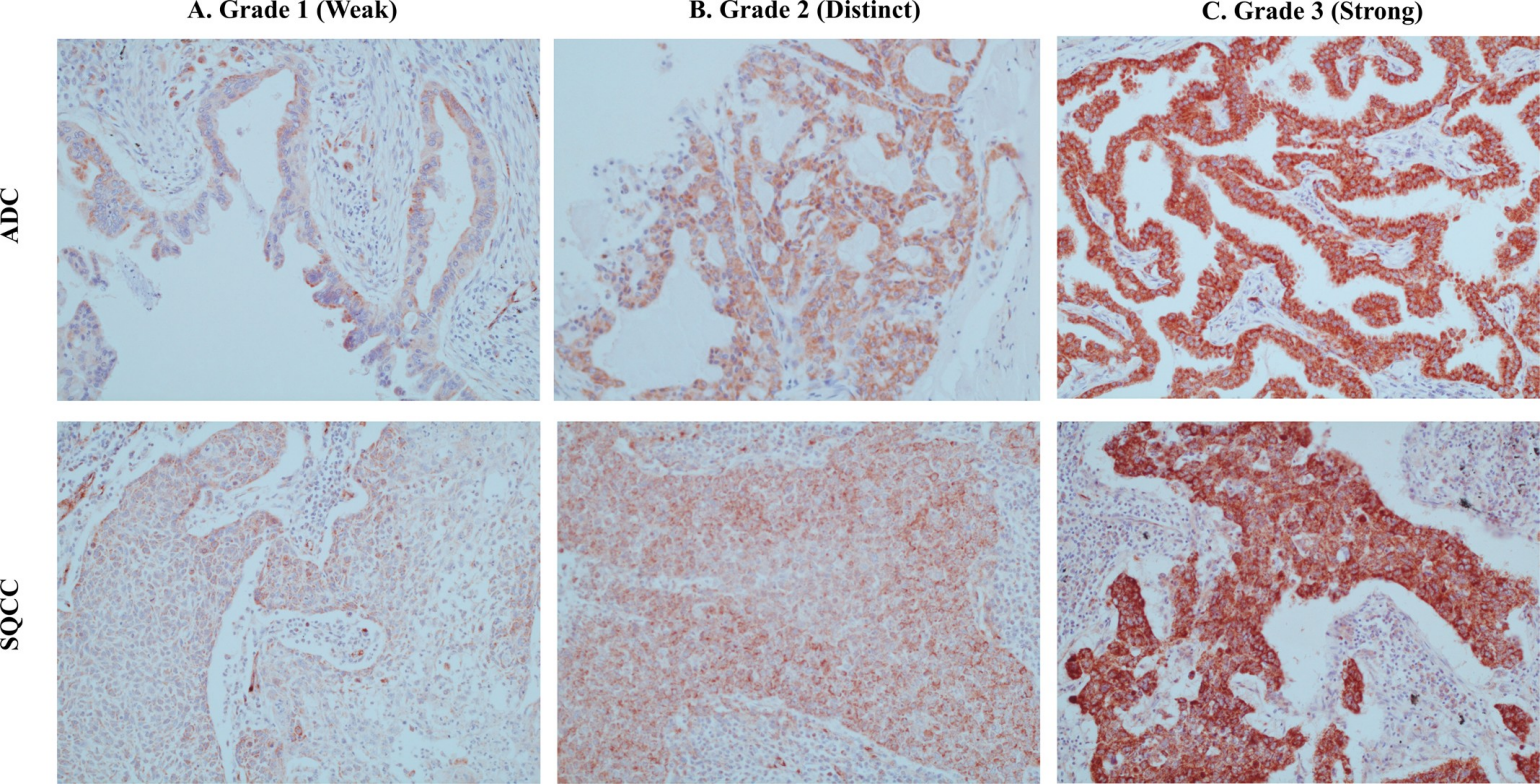
Representative examples of immunohistochemical staining for romo1 with different histologic scores (H scores).

The expression of romo1 in NSCLC was analyzed by immunohistochemical staining using the LABS® 2 System (Dako, Carpinteria, CA, USA) according to the manufacturer’s instructions. Sections were deparaffinized, rehydrated, immersed in an H_2_O_2_ and methanol solution, and incubated overnight with primary antibodies against romo1. Incubations were performed in antibody diluent (Dako) at dilutions of 1:200. Sections were incubated for 10 min with a biotinylated linker and processed by avidin/biotin immunohistochemistry. 3,3′-Diaminobenzidine (DAB) was used as a chromogen in conjunction with the Liquid DAB Substrate Kit (Novocastra, Newcastle upon Tyne, UK). Romo1 expression was independently evaluated by two pathologists (YJ Cha and JH Park) who were blinded clinical information. If they scored differently, peer review with an independent pathologist was conducted. After sufficient discussion, a final score was given. Sections were examined under a light microscope at 200× magnification, and cytoplasmic staining was considered to be positive for romo1 expression. Romo1 expression was scored according to the staining intensity and percentage of positive cells. Staining intensity was classified as follows: 0, no staining; 1, weak; 2, distinct; or 3, strong (Fig 1). Quantification of positivity (0–100%) was an estimated percentage of tumor cells with the specific staining intensity. The final histologic scores (H scores) were calculated by multiplying staining intensities and the proportion of tumor cells with each staining intensity calculated by the following equation: H score = (proportion of tumor cells with no staining × 0) + (proportion of tumor cells with weak intensity × 1) + (proportion of tumor cells with distinct intensity × 2) + (proportion of tumor cells with strong intensity × 3). The H scores range from 0 to 300. For example, the right and lower figure in Fig 1, we can calculate H score as follow: (50% × 0) + (0% × 1) + (0% × 2) + (50% × 3) = 150. The H scores range from 0 to 300. The cut-off H score for discriminating between low and high romo1 expression was defined as the median H score. The median H score obtained in this study was 150. This cut-off value is similar to that used in a previous study [15].

### Statistical analysis

The proportions of low and high H scores in different patient groups were analyzed by the Chi-square test or Fisher’s exact test, as appropriate. Clinical outcomes, including disease-free survival (DFS) and overall survival (OS), were assessed. DFS was defined as time from surgical biopsy to recurrence or death, and OS was the time from surgical biopsy to death from any cause. Data for patients without tumor recurrence or death were censored at the time of last follow-up. Survival curves were generated using the Kaplan–Meier method. *P-*values of less than 0.05 were considered significant. Associations between clinical parameters and survival were first evaluated by a univariate analysis using the log rank test. Subsequently, a multivariate Cox’s proportional hazard regression analysis was conducted with adjustment for parameters with *P*-values of less than 0.05 in the univariate analysis. With regards to patient N stage, because the distribution between the N0 and N1 or higher groups showed a large deviation, the result of univariate analysis was less than 0.05, and N stage was not included in the multivariate analysis. Statistical analyses were implemented in SPSS version 23.0 for Windows (SPSS, Chicago, IL, USA).

## Results

### Clinical characteristics of patients according to romo1 expression

A total of 98 patients were enrolled. Clinical characteristics according to romo1 expression and representative images of immunohistochemical staining for romo1 are shown in Fig 1. Romo1 was primarily localized to the cytoplasm of cancer cells, regardless of type of NSCLC. The romo1 H scores were normally distributed with a mean of 149 ± 8.60. The clinical characteristics for each group are summarized in Table 1. Using the median H score of 150 as a threshold, 53 patients (54%) were assigned to the high romo1 expression group and 45 patients (46%) were assigned to the low romo1 expression group. The median age was 64 years (range 32–78 years), and 64 patients (65%) were male. Age, sex, and smoking status were not significantly different between the two groups. Heavy smoker (>20 pack-year) was slightly more prevalent in the low romo1 expression group (60%) than in the high romo1 expression group (41%); however, this difference was not statistically significant (*P* = 0.065). With respect to the pathologic type, squamous cell type was slightly more prevalent in the low romo1 expression group (53%) than in the high romo1 expression group (36%), but this difference was not significant (*P* = 0.197). The distribution of T stages was not significantly different between the two groups. However, an advanced N stage was more frequently observed in the high romo1 expression group (N1, 47%; N2, 25%) than in the low romo1 expression group (N1, 24%; N2, 18%). N0 stage was more frequent in the low romo1 expression group (58%) than in the high romo1 expression group (28%). An advanced final stage of NSCLC was more frequent in the high romo1 expression group (I, 21%; II 45%; III, 34%) than in the low romo1 expression group (I, 42%; II, 40%; III, 18%) (*P* = 0.044). In addition, all patients (97/98) except one, underwent radical resection surgery. Forty percent of subjects in the low romo1 group and 41% of those in the high romo1 group received adjuvant platinum-based chemotherapy (*P* = 1.000). Seven percent of patients in the low romo1 group and 13% in the high romo1 group were treated with chemo/radio-therapy (*P* = 1.000) (Table 1).

**Table 1:** Clinical characteristics of patients according to low and high romo1 expression.

Variables	Romo1 expression		Total	*P-*value
	Low (H score <150)	High (H score ≥150)		
All	45(100)	53(100)	98	
Age, years				0.476
≤65	27 (60)	28 (53)	55	
>65	18 (40)	25 (47)	43	
Sex				0.124
Female	12 (27)	22 (42)	34	
Male	33 (73)	31 (59)	64	
Smoking status				0.773
Never	15 (35)	20 (38)	35	
Ever	28 (65)	33 (62)	61	
Smoking pack-year				0.065
≤20	17 (40)	31 (59)	48	
>20	26 (60)	22 (41)	48	
Underlying lung ds				0.281
None	32 (78)	45 (87)	77	
Present	9 (22)	7(14)	16	
Pathology				0.224
ADC	19 (42)	31 (59)	50	
SQC	24 (53)	19 (36)	434	
Others^#^	2 (4)	3 (6)	5	
T stage				0.558
T1	9 (20)	7 (13)	16	
T2	27 (60)	33 (62)	60	
T3	8 (18)	9 (17)	17	
T4	1 (2)	4 (8)	5	
N stage				0.011
N0	26 (58)	15 (28)	41 40	
N1	11 (24)	25 (47)	36 38	
N2	8 (18)	13 (25)	21 19	
Overall stage				0.044
I	19 (42)	11 (21)	30	
II	18 (40)	24 (45)	42	
III	8 (18)	18 (34)	26	
Surgery				0.537
Wedge resection	1 (2)	0 (0)	1	
Lobectomy	37 (82)	45(85)	82	
Pneumonectomy	7 (16)	8 (15)	15	
Adjuvant chemotherapy				
No	27 (60)	31 (59)	58	1.000
Yes	18 (40)	22 (41)	40	
Chemo/Radiotherapy				
No	42 (93)	46 (87)	88	1.000
Yes	3 (7)	7 (13)	10	

Values are presented as n (%)

Abbreviations: ADC, adenocarcinoma; SQC, squamous cell carcinoma

# Other types included large cell lung cancer (3 cases) and unknown NSCLC type (1 case)

### Distribution of romo1 expression levels according to stage and lymph node metastasis

**Fig 2:**
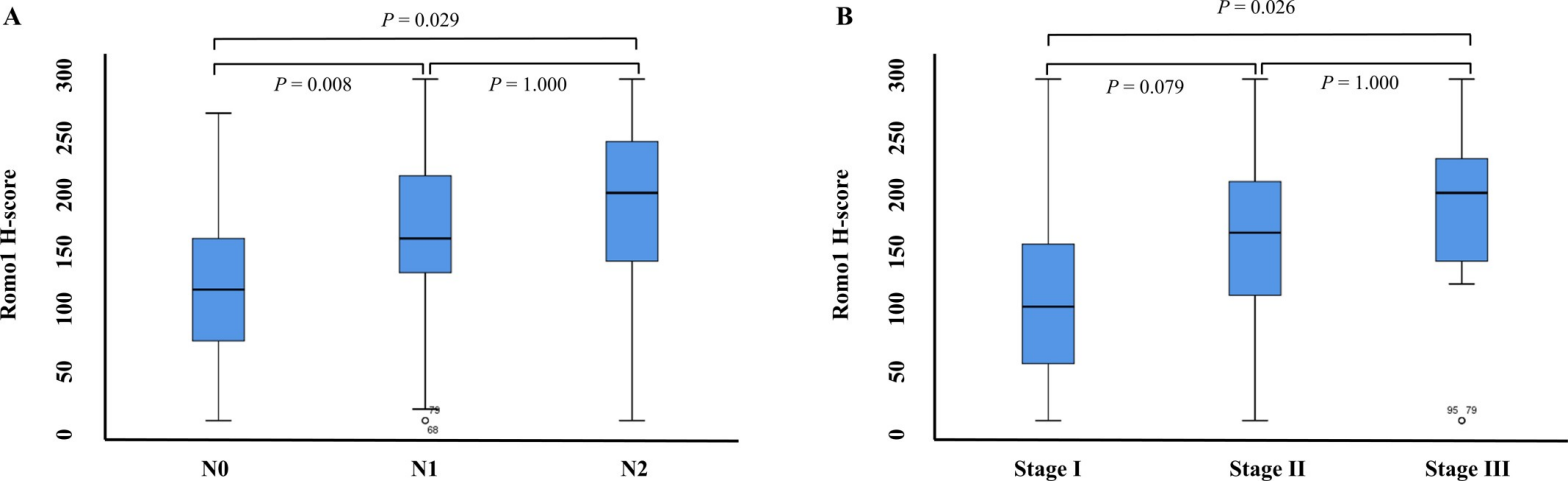
Relationship between Romo1 expression and (A) N stage and (B) Overall stage. A comparison of the romo1 H-score according to the stage was performed by Mann-Whitney test and Wilcoxon rank-sum test, and the p-value are stated above the box plot. The box signifies lower (Q1) and upper (Q3) quartiles, and the median is represented by a short black line within the box.

A differential distribution of romo1 expression was observed among patients with different T stages (mean H score ± SEM; T1: 124 ± 24.8, T2: 155 ± 10.9, T3: 144 ± 14.0, and T4: 182 ± 54.7; *P* = 0.312). The romo1 expression was significantly lower in patients with N0 disease (mean H score: 116 ± 10.6) than in those with N1 (mean H score: 173 ± 14.0; *P* = 0.008) or N2 disease (173 ± 21.7; *P* = 0.029). The stage III group showed significantly higher romo1 expression (173 ± 16.7) than that in the stage I group (114 ± 14.0, *P* = 0.026) (Fig 2).

In addition, we compared the distribution of romo1 expression intensities among groups classified by stage. Although a correlation was not detected for T stage, there were significant associations of the distribution of romo1 expression intensity with N stage and total stage.

The area (%) of grade 1 expression was significantly smaller in subjects without lymph node metastasis than in subjects with lymph node metastasis (N0: 37.0 ± 5.22 vs. ≥N1: 19.5 ± 2.90; *P* = 0.005). The area (%) of grade 3 expression was significantly larger in subjects with lymph node metastasis than in subjects without lymph node metastasis (N0: 6.00 ± 2.88 vs. ≥N1: 27.9 ± 4.73; *P* < 0.001). Total stage showed the same pattern, with statistical significance (Table 2).

**Table 2:** Relationship between Romo1 expression intensity and stage.

Variables			Romo1 expression intensity (area %)						
		Grade 0	p-value	Grade 1	p-value	Grade 2	p-value	Grade 3	p-value
All (%)	98(100)	N(%)							
**T stage**			0.572		0.689		0.255		0.713
T1	17 (17)	3(37.5)		22.4 ± 7.10		30.6 ± 8.29		15.3 ± 8.0	
T2	59 (60)	3(37.5)		26.9 ± 3.38		30.5 ± 3.77		21.9 ± 4.2	
T3	17 (17)	1(12.5)		35.3 ± 8.75		43.5 ± 7.71		7.06 ± 4.18	
T4	5 (5)	1(12.5)		8.0 ± 5.83		30.0 ± 16.7		38.0 ± 23.3	
**N stage**			0.883		**0.005**		0.419		**<0.001**
N0	40 (41)	3(37.5)		37.0 ± 5.22		29.8 ± 4.84		6.00 ± 2.88	
≥N1	58 (59)	5(62.5)		19.5 ± 2.90		34.8 ± 4.06		27.9 ± 4.73	
**Overall stage**					**0.003**		0.057		**0.042**
I	30 (31)	2(25.0)	0.883	36.7 ± 5.58		23.3 ± 4.68		9.33 ± 4.26	
II	42 (43)	3(37.5)		26.7 ± 4.45		35.5 ± 5.01		21.4 ± 5.34	
III	26 (27)	3(37.5)		15.0 ± 3.89		39.2 ± 6.22		26.2 ± 6.68	

### Correlation of romo1 expression with survival measures

**Fig 3:**
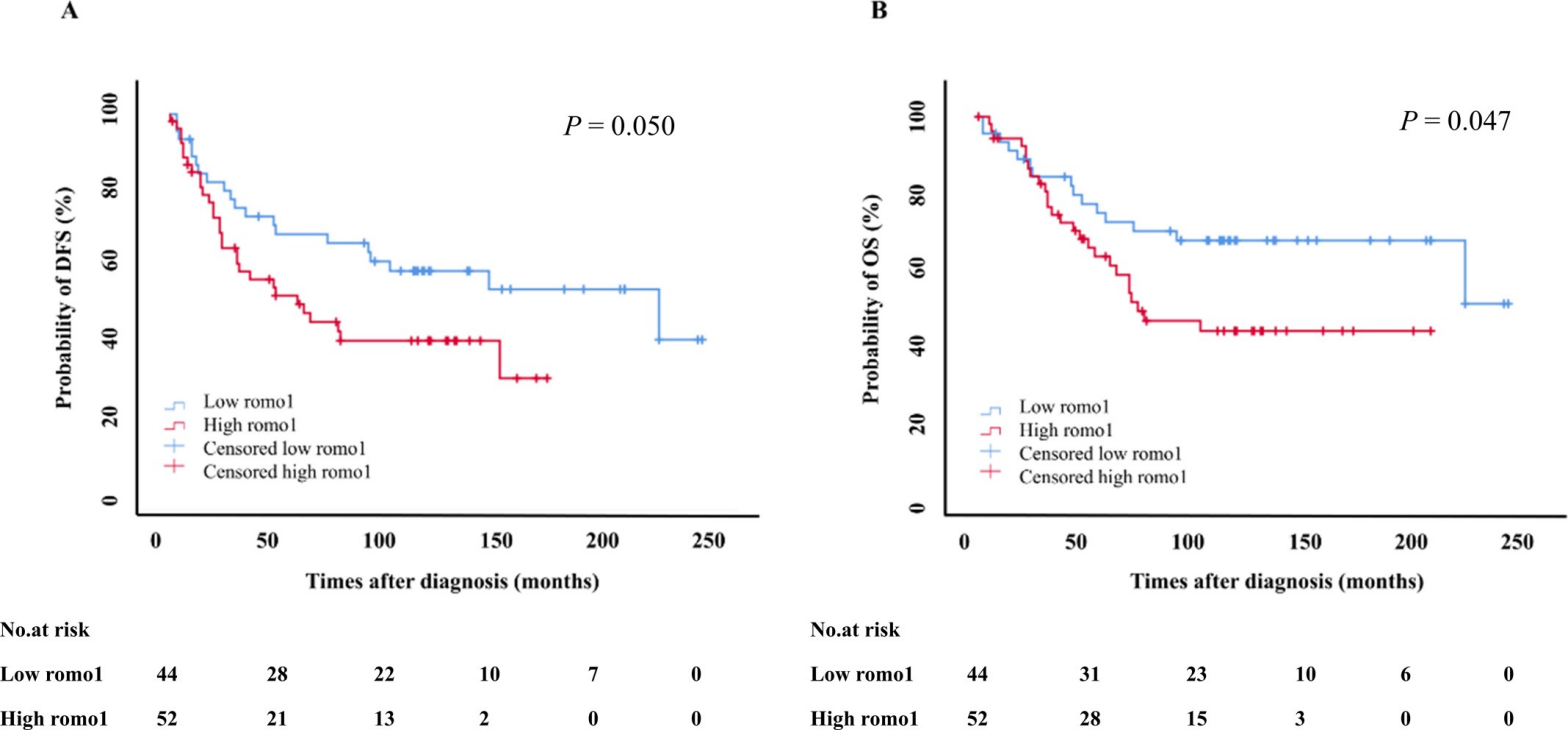
Kaplan–Meier survival curves for disease free survival (DFS) (A) and overall survival (OS) (B) in the overall population. *P*-values were determined using the log-rank test. romo1, reactive oxygen species modulator 1.

The prognostic significance of romo1 expression was analyzed in 98 NSCLC patients. Among them, 45 (45.9%) had low romo1 expression and 53 (54.1%) had high romo1 expression. The DFS and OS of patients were analyzed divided to low romo1 group and high romo1 group (Fig 3). The mean DFS was 129 ± 11.3 months (95% confidence interval [CI], 107–151 months). According to the Kaplan-Meier survival curves, the high romo1 group had shorter DFS than the low romo1 group, with marginal significance (85.4±10.4 vs. 152±15.9 months, P = 0.050, log-rank test, Fig 3A). The mean OS was 152±10.9 months (95% CI, 131–174 months). Although statistically not significant, the OS of high romo1 group was shorter than that of low romo1 group (117±12.5 vs. 175±15.1 months, *P* = 0.047, log-rank test, Fig 3B).

**Fig 4:**
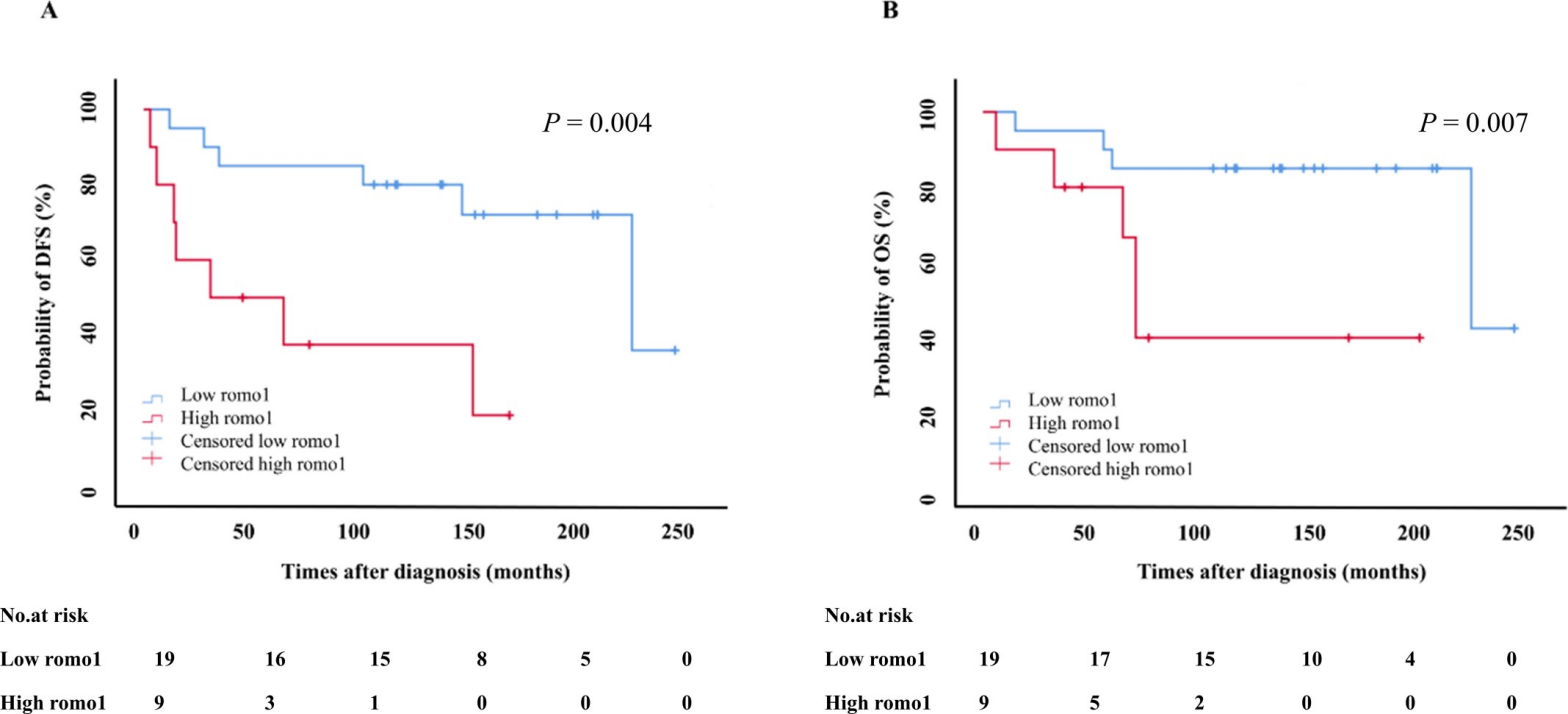
Kaplan–Meier survival curves for disease free survival (DFS) (A) and overall survival (OS) (B) in stage I patients. *P*-values were determined using the log-rank test. romo1, reactive oxygen species modulator 1.

A subgroup analysis was performed according to the stage. In stage I patients (n = 30), the high romo1 group had significantly shorter DFS and OS than the low romo1 group (DFS: 75.6±22.4 vs. 191±18.8 months, *P* = 0.004; OS: 113±26.5 vs. 208±16.7 months, *P* = 0.007, log-rank test, Fig 4). However, in the subgroup analysis of stage II (n = 42) and III (n = 26) patients, there was no significant difference in the DFS and OS for the high and low romo1 groups. (Stage II: DFS 75.7±10.5 vs. 144±25.9 months, *P* = 0.478; OS 84.9±9.7 vs. 157±25.5 months, *P* = 0.461; Stage III: DFS 69.3±17.0 vs. 38.0±16.2 months, *P* = 0.416; OS 111±22.4 vs. 57.9±17.7 months, *P* = 0.646, log-rank test). Interestingly, the expression of romo1 was associated with poor prognosis, especially in stage I NSCLC patients.

The univariate and multivariate Cox-regression analyses were performed to determine the prognostic factors in the overall stage of patients (Table 3). In the univariate analysis, presence of lymph node metastasis, overall stage, treatment with platinum-based chemotherapy, and romo1 expression were found to be associated with poor DFS (*P* = 0.012, *P* = 0.004, *P* = 0.001 and *P* = 0.054). However, in the multivariate analysis, only treatment with platinum-based chemotherapy showed a significant association with poor DFS (*P* = 0.001). In stage I patients, univariate analysis showed that age, treatment with platinum-based chemotherapy, and romo1 expression had a significant impact on DFS (Table 4) (*P* = 0.032, *P* < 0.001 and *P* = 0.004). In the multivariate analysis, age, platinum-based chemotherapy and high romo1 were significant predictors of DFS (*P* = 0.043, *P* = 0.005 and *P* = 0.009). There was no significant correlation between the level of expression of romo1 in stage II or III (S1 and S2 Tables).

**Table 3:** Survival analyses results according to clinical parameters of all subjects.

Variables		Number (%)	DFS					OS				
			Univariate analysis			Multivariate analysis		Univariate analysis			Multivariate analysis	
			Mean DFS (months)	adjusted HR (95%CI)	p-value	adjusted HR (95%CI)	p-value	Mean OS (months)	adjusted HR (95%CI)	p-value	adjusted HR (95%CI)	p-value
Age, years	≤65	55(56)	145±15.0	Ref	0.122	NA		166±13.8	Ref	0.122	NA	
	>65	43(44)	86.4±11.2	1.55(0.88–2.72)				118±14.3	1.68(0.91–3.11)			
Sex	Female	34(35)	137±18.8	Ref	0.699	NA		154±17.9	Ref	0.973	NA	
	Male	64(65)	118±15.1	1.12(0.62–2.02)				143±12.3	0.97(0.52–1.80)			
Smoking, pys	≤20	48(50)	122±15.8	Ref	0.710	NA		149±15.0	Ref	0.969	NA	
	>20	48(50)	117±13.7	0.89(0.51–1.58)				134±13.4	0.95(0.52–1.74)			
Pathology	ADC	50(51)	119±14.9	Ref	0.474	NA		150±14.6	Ref	0.882	NA	
	SQCC	43(44)	147±17.3	0.92(0.56–1.50)				157±16.8	0.90(0.48–1.70)			
T Stage	T1	17(17)	97.7±21.7	Ref	0.433	NA		134±21.6	Ref	0.954	NA	
	≥T2	81(83)	134±12.5	0.83(0.12–1.66)				153±12.0	1.17(0.52–2.63)			
N stage	N0	40(41)	161±16.1	Ref	0.012	Ref	0.230	179±14.7	Ref	0.026	Ref	0.697
	≥N1	58(59)	78.6±9.11	2.13(1.16–3.93)		1.52(0.80–2.88)		118±12.5	1.85(0.67–3.54)		1.27(0.55–2.92)	
Overall stage	I	30(31)	164 ± 18.2	Ref	0.004	Ref	0.935	190±16.3	Ref	0.018	Ref	0.058
	≥II	68(69)	117 ± 13.5	1.77(0.93–3.37)		1.00(0.42–2.41)		135±13.3	1.93(0.95–3.94)		2.14(0.98–4.69)	
Platinum-based	No	58(59)	160±14.3	Ref	0.001	Ref	0.001	176±13.6	Ref	0.007	Ref	0.029
chemotherapy	Yes	40(41)	62.0±8.41	0.64(0.48–0.86)		2.64(1.47–4.76)		80.0±8.03	2.14(1.15–3.97)		2.14(0.98–3.86)	
Radiotherapy	No	88(90)	127±12.2	Ref	0.326	NA		152±11.8	Ref	0.613	NA	
	Yes	10(10)	71.0±23.3	1.53(0.65–3.61)				94.2±20.4	1.27(0.49–3.25)			
Romo1	Low	45(46)	152 ± 15.9	Ref	0.050	Ref	0.062	175±15.1	Ref	0.047	Ref	0.149
	High	53(54)	85.4 ± 10.4	1.77(0.99–3.16)		1.74(0.97–3.11)		117±12.5	1.81(0.96–3.43)		1.64(0.38–3.21)	

DFS: disease free survival; OS: overall survival, HR: hazard ratio, CI: confidence interval; pys: pack-years

**Table 4:** Survival analyses results according to clinical parameters of stage I (n = 30).

Variables		number (%)	DFS					OS				
			Univariate analysis			Multivariate analysis		Univariate analysis			Multivariate analysis	
			Mean DFS (months)	adjusted HR (95%CI)	p-value	adjusted HR (95%CI)	p-value	Mean OS (months)	adjusted HR (95%CI)	p-value	adjusted HR (95%CI)	p-value
Age, years	≤65	17(57)	193±20.1	Ref	0.032	Ref	0.043	217±14.9	Ref	0.030	Ref	0.038
	>65	13(43)	88.9±20.0	4.58(1.34–15.6)		7.79(1.07–56.7)		130±24.1	4.66(1.15–18.9)		5.79 (1.10–30.4)	
Sex	Female	13(43)	173±27.3	Ref	0.525	NA		175±26.4	Ref	0.345	NA	
	Male	17(57)	136±20.5	1.51(0.49–4.66)				181±14.6	0.50(0.14–1.79)			
Smoking, pys	≤20	18(63)	147±23.7	Ref	0.641	NA		180±21.9	Ref	0.517	NA	
	>20	11(37)	149±25.8	0.75(0.23–2.42)				182±18.4	0.59(0.15–2.31)			
Pathology	ADC	16(59)	175±20.7	Ref	0.689	NA		202±19.1	Ref	0.498	NA	
	SQCC	11(41)	140±28.3	1.34(0.37–4.81)				163±22.6	1.65(0.41–6.60)			
T Stage	T1	9(30)	126±27.5	Ref	0.363	NA		163±28.5	Ref	0.720	NA	
	≥T2	21(70)	168±22.0	0.64(0.21–1.96)				193±18.2	0.96(0.25–3.72)			
N stage	N0	27(90)	171±19.0	Ref	0.027	NA		194±16.34	Ref	0.262	NA	
	≥N1	3(10)	65.3±44.2	3.66(0.98–13.6)				118±60.1	4.42(0.90–21.7)			
Platinum-based	No	21(70)	196±17.5	Ref	<0.001	Ref	0.005	208±16.7	Ref	0.048	Ref	0.047
chemotherapy	Yes	9(30)	48.4±14.8	8.61(2.19–33.9)		7.90(1.88–33.2)		94.7±13.3	3.83(1.01–14.6)		4.61(1.02–20.9)	
Romo1	Low	20(67)	191±18.8	Ref	0.004	Ref	0.009	208±16.7	Ref	0.007	Ref	0.294
	High	10(33)	75.6±22.4	5.82(1.75–19.3)		5.59(1.54–20.3)		113±26.5	3.68(0.96–14.0)		2.67(0.43–16.8)	

DFS: disease free survival; OS: overall survival, HR: hazard ratio, CI: confidence interval; pys: pack-years

## Discussion

We analyzed romo1 expression in lung specimens obtained from subjects who underwent surgical biopsy to evaluate its prognostic value in the early stage of NSCLC. Our results indicated that the overexpression of romo1 is significantly associated with lymph node metastasis and advanced stage. It could also independently predict poor survival in subjects especially in those with early-stage NSCLC who underwent surgical biopsy. Previous studies have demonstrated that old age, heavy smoking history, and advanced stage are important poor prognostic markers for NSCLC [9]. Although above factors were not significant in our study, we identified the overexpression of romo1 is an independent prognostic factor for NSCLC. Romo1 expression can be easily checked using specimens obtained by surgical biopsy. Immunohistochemical staining for romo1 and observations by light microscopy do not require excessive costs, effort, or time. Accordingly, this new marker will be useful to predict prognosis, independent of age, smoking status, and stage.

The overexpression of romo1 predicted the presence of lymph node metastasis in this study. Especially, we also found that the percentage area of romo1 expression intensity is significantly different according to the lymph node metastasis, for the first time. Recently, romo1 overexpression has been associated with lymphatic invasion of lung cancer and a poor prognosis. The VEGF gene family includes signaling proteins that induce angiogenesis and lymphangiogenesis [12], and romo1 is a regulator of ROS associated with the VEGF family [13]. It has been suggested that romo1 may be associated with lymphatic metastasis via ROS and VEGF signaling based on observations of a significant correlation between romo1 expression and VEGF-C and ROS in lung cancer tissues [14]. Therefore, the correlation between the H score of romo1 and N stage is consistent with the previous studies suggesting that romo1 is associated with lymphatic metastasis [14].

We showed that the expression of romo1 increased significantly according to the overall stage in patients with NSCLC (Fig 2). Studies have shown that romo1 expression is a poor prognostic marker in early stage (stage I–II) patients who underwent surgical resection and in advanced stage (stage IIIB or higher) patients who underwent palliative chemotherapy [2, 15]. However, our study enrolled patients with stage I to III cancer, based on surgical biopsies, and our findings suggest that romo1 expression increases with cancer stage and may be a factor associated with poor prognosis. Although the mechanism how romo1 is associated with poor prognosis in NSCLC cannot be fully explained, increased tumor invasion by romo1 provides a possible explanation. Oxidative stress is associated with complex processes involved in cancer progression, including migration, invasion, angiogenesis, and metastasis [13]. Romo1 produces ROS by oxidative stress and eventually causes cell hyperplasia and cancer cell invasion. A recent study has demonstrated that romo1 overexpression is related to vascular invasion in patients with hepatocellular carcinoma [5] and lymph node metastasis [14]. These findings suggest that the overexpression of romo1 leads to cancer cell proliferation, vascular invasion, and lymph node metastasis, which may explain the poor prognosis.

Our data also show that the expression of romo1 was related to prognosis, especially in early NSCLC. Particularly in stage I, romo1 overexpression was significantly related to both progression and survival. For stage I NSCLC, the conventionally treatment option is surgical resection, and adjuvant chemotherapy is selected depending on stage (IA or IB). Platinum-based chemotherapy in stage IA is related to a harmful prognosis [16], and post-operative treatment in stage IB remains controversial. National Comprehensive Cancer Network (NCCN) guidelines consider observation or adjuvant chemotherapy appropriate options for patients with resected stage IB NSCLC, depending on risk factors for recurrence [17]. Therefore, romo1 is a potentially useful predictor of prognosis, especially in the early stage of NSCLC. Considering that romo1 is associated with the progression, invasiveness, and metastasis of cancer, it is also a potential molecular target for future cancer therapy. Further studies of the mechanism by which romo1 is involved in cancer progression and invasiveness are required to demonstrate that the inhibition of romo1 is a potential therapeutic strategy.

In our data, the mean romo1 H score in stage I was lower, with a larger standard deviation than those at other stages therefore, it is presumed that there was discrimination in prediction of prognosis. However, a previous study has shown that romo1 is a poor prognostic factor in advanced NSCLC [15]; additional patient data analyzed according to stage may further support its use as a prognostic indicator in advanced NSCLC.

This study had several limitations. First, it was performed retrospectively at a single institution with a relatively small sample size. Second, we performed semiquantitative immunohistochemical analyses. However, a standard quantification method for romo1 expression has not been established. Third, our results may be affected by selection bias because only patients who were eligible for surgical biopsy were enrolled.

## Conclusion

Using data from NSCLC subjects who underwent surgical biopsy, we demonstrated that the expression of romo1 increases according to stage, especially in the presence of lymph node metastasis. The distribution of romo1 expression intensities was significantly different according to lymph node metastasis. In addition, the overexpression of romo1 showed a significant association with poor prognosis, especially in stage I NSCLC.

## Supporting information

S1 Table Survival analyses according to clinical parameters for stage II. (DOCX)

S2 Table Survival analyses according to clinical parameters for stage III. (DOCX)

S1 Fig Scatter plot illustrating the H-score of romo1 expression each individual. (TIF)

## References

[1] Chung YM, Kim JS, Do Yoo Y. A novel protein, Romo1, induces ROS production in the mitochondria. Biochemical and biophysical research communications. 2006;347(3):649–55. 10.1016/j.bbrc.2006.06.140 16842742

[2] Hwang IT, Chung YM, Kim JJ, Chung JS, Kim BS, Kim HJ, et al Drug resistance to 5-FU linked to reactive oxygen species modulator 1. Biochemical and biophysical research communications. 2007;359(2):304–10. 10.1016/j.bbrc.2007.05.088 17537404

[3] Martindale JL, Holbrook NJ. Cellular response to oxidative stress: signaling for suicide and survival. J Cell Physiol. 2002;192(1):1–15. 10.1002/jcp.10119 . 12115731

[4] Chung YM, Kim JS, Yoo YD. A novel protein, Romo1, induces ROS production in the mitochondria. Biochem Biophys Res Commun. 2006;347(3):649–55. 10.1016/j.bbrc.2006.06.140 . 16842742

[5] Chung JS, Park S, Park SH, Park ER, Cha PH, Kim BY, et al Overexpression of Romo1 promotes production of reactive oxygen species and invasiveness of hepatic tumor cells. Gastroenterology. 2012;143(4):1084–94. e7. 10.1053/j.gastro.2012.06.038 22749933

[6] Kim HJ, Jo MJ, Kim BR, Kim JL, Jeong YA, Na YJ, et al Reactive oxygen species modulator-1 (Romo1) predicts unfavorable prognosis in colorectal cancer patients. PloS one. 2017;12(5):e0176834 10.1371/journal.pone.0176834 28472059 PMC5417558

[7] Stampfli MR, Anderson GP. How cigarette smoke skews immune responses to promote infection, lung disease and cancer. Nat Rev Immunol. 2009;9(5):377–84. 10.1038/nri2530 . 19330016

[8] Lawless MW O 'Byrne KJ, Gray SG. Oxidative stress induced lung cancer and COPD: opportunities for epigenetic therapy. J Cell Mol Med. 2009;13(9A):2800–21. 10.1111/j.1582-4934.2009.00845.x 19602054 PMC4498937

[9] Kim HC, Jung CY, Cho DG, Jeon JH, Lee JE, Ahn JS, et al Clinical characteristics and prognostic factors of lung cancer in Korea: a pilot study of data from the Korean nationwide lung cancer registry. Tuberculosis and respiratory diseases. 2019;82(2):118–25. 10.4046/trd.2017.0128 29926550 PMC6435924

[10] Karaman S, Detmar M. Mechanisms of lymphatic metastasis. The Journal of clinical investigation. 2014;124(3):922–8. 10.1172/JCI71606 24590277 PMC3938272

[11] Goldstraw P. The 7th Edition of TNM in Lung Cancer: what now? Journal of Thoracic Oncology. 2009;4(6):671–3. 10.1097/JTO.0b013e31819e7814 19461399

[12] Ushio–Fukai M. VEGF signaling through NADPH oxidase-derived ROS. Antioxidants & redox signaling. 2007;9(6):731–9. 10.1089/ars.2007.1556 17511588

[13] Wu W-S. The signaling mechanism of ROS in tumor progression. Cancer and Metastasis Reviews. 2006;25(4):695–705. 10.1007/s10555-006-9037-8 17160708

[14] Kim HJ, Jo MJ, Kim BR, Kim JL, Jeong YA, Na YJ, et al Overexpression of romo1 is an unfavorable prognostic biomarker and a predictor of lymphatic metastasis in non-small cell lung cancer patients. OncoTargets and therapy. 2018;11:4233 10.2147/OTT.S161587 30087567 PMC6061672

[15] Lee SH, Choi SI, Lee JS, Kim CH, Jung WJ, Lee EJ, et al Reactive oxygen species modulator 1 (Romo1) predicts poor outcomes in advanced non-small cell lung cancer patients treated with platinum-based chemotherapy. Cancer research and treatment: official journal of Korean Cancer Association. 2017;49(1):141 10.4143/crt.2016.133 27188201 PMC5266402

[16] Pignon J-P, Tribodet H, Scagliotti GV, Douillard J-Y, Shepherd FA, Stephens RJ, et al Lung adjuvant cisplatin evaluation: a pooled analysis by the LACE Collaborative Group. Journal of clinical oncology. 2008;26(21):3552–9. 10.1200/JCO.2007.13.9030 18506026

[17] Ettinger DS, Aisner DL, Wood DE, Akerley W, Bauman J, Chang JY, et al NCCN guidelines insights: non–small cell lung cancer, version 5.2018. Journal of the National Comprehensive Cancer Network. 2018;16(7):807–21. 10.6004/jnccn.2018.0062 30006423

