# Supplementary material for: Reactive oxygen species modulator 1 expression predicts lymph node metastasis and survival in early-stage non-small cell lung cancer
Source: PLoS One. 2020 Dec 1;15(12):e0239670. doi: 10.1371/journal.pone.0239670 (PMC7707601; doi:10.1371/journal.pone.0239670)
Supplement: S1 Table — (DOCX) [file pone.0239670.s001.docx]

**S1 Table. Survival analyses results according to clinical parameters of stage II (n=42)**

| Variables | | Number  (%) |  | DFS | | | | |  | |  | OS | | | |
| --- | --- | --- | --- | --- | --- | --- | --- | --- | --- | --- | --- | --- | --- | --- | --- |
|  |  |  |  | Univariate analysis | | | Multivariate analysis | | | |  | Univariate analysis | | Multivariate analysis | |
|  |  |  | Mean DFS(months) | | adjusted HR  (95%CI) | p-value | adjusted HR  (95%CI) | p-value | | Mean  OS(months) | | adjusted HR  (95%CI) | p-value | adjusted HR  (95%CI) | p-value |
| Age, years | ≤65 | 21(50) | 155±22.8 | | Ref | 0.161 | Ref | 0.420 | | 174±21.6 | | Ref | 0.043 | Ref | 0.071 |
|  | >65 | 21(50) | 66.2±10.3 | | 1.86(0.78-4.45) |  | 1.47(0.58-3.75) |  | | 68.4±9.6 | | 2.63(1.03-6.72) |  | 2.01(0.74-5.45) |  |
| Sex | Female | 12(29) | 125±32.8 | | Ref | 0.786 | Ref | 0.868 | | 132±30.6 | | Ref | 0.698 | Ref | 0.657 |
|  | Male | 30(69) | 125±18.1 | | 0.88(0.34-2.26) |  | 0.89(0.22-3.54) |  | | 138±18.0 | | 0.83(0.31-2.18) |  | 0.71(0.16-3.25) |  |
| Smoking, pys | ≤20 | 18(43) | 120±25.4 | | Ref | 0.620 | Ref | 0.265 | | 141±24.0 | | Ref | 0.979 | Ref | 0.121 |
|  | >20 | 23(57) | 82.4±10.4 | | 0.81(0.34-1.89) |  | 2.69(0.47-15.4) |  | | 86.2±10.3 | | 1.01(0.41-2.49) |  | 5.19(0.65-41.6)) |  |
| Pathology | ADC | 19(45) | - | | Ref | 0.241 | Ref | 0.110 | | - | | Ref | 0.519 | Ref | 0.091 |
|  | SQCC | 22(55) | - | | 0.59(0.25-1.41) |  | 0.29(0.06-1.32) |  | | - | | 0.74(0.30-1.83) |  | 0.22(0.04-1.27) |  |
| T Stage | T1 | 4(10) | 55.7±17.0 | | Ref | 0.676 | Ref | 0.648 | | 89.3±15.2 | | Ref | 0.529 | Ref | 0.702 |
|  | ≥T2 | 38(90) | 133±17.7 | | 0.73(0.17-3.16) |  | 0.67(0.12-3.77) |  | | 140±17.4 | | 1.91(0.26-14.3) |  | 1.57(0.16-15.6) |  |
| N stage | N0 | 12(29) | 125±29.1 | | Ref | 0.945 | Ref | 0.699 | | 131±27.6 | | Ref | 0.725 | Ref | 0.668 |
|  | ≥N1 | 30(69) | 81.0±9.86 | | 0.97(0.39-2.41) |  | 0.83(0.31-2.19) |  | | 89.9±9.4 | | 0.85(0.33-2.15) |  | 0.74(0.27-2.02) |  |
| Platinum-based | No | 25(60) | 141±22.6 | | Ref | 0.520 | Ref | 0.975 | | 152±22.1 | | Ref | 0.598 | Ref | 0.995 |
| chemotherapy | Yes | 17(40) | 77.2±11.5 | | 1.33(0.56-3.13) |  | 0.98(0.38-2.53) |  | | 86.3±10.8 | | 1.28(0.52-3.15) |  | 0.80(0.29-2.18) |  |
| Romo1 | Low | 17(40) | 144 ± 25.9 | | Ref | 0.478 | Ref | 0.634 | | 157±25.5 | | Ref | 0.461 | Ref | 0.567 |
|  | High | 25(60) | 75.7 ± 10.5 | | 1.38(0.57-3.34) |  | 1.29(0.45-3.77) |  | | 84.9±9.7 | | 1.42(0.56-3.63) |  | 1.40(0.44-4.42) |  |

DFS: disease free survival; OS: overall survival, HR: hazard ratio, CI: confidence interval; pys: pack-years
