# Supplementary material for: Reactive oxygen species modulator 1 expression predicts lymph node metastasis and survival in early-stage non-small cell lung cancer
Source: PLoS One. 2020 Dec 1;15(12):e0239670. doi: 10.1371/journal.pone.0239670 (PMC7707601; doi:10.1371/journal.pone.0239670)
Supplement: S2 Table — (DOCX) [file pone.0239670.s002.docx]

**S2 Table. Survival analyses results according to clinical parameters of stage III (n=26)**

| Variables | | Number  (%) |  | DFS | | | | |  | |  | OS | | | |
| --- | --- | --- | --- | --- | --- | --- | --- | --- | --- | --- | --- | --- | --- | --- | --- |
|  |  |  |  | Univariate analysis | | | Multivariate analysis | | | |  | Univariate analysis | | Multivariate analysis | |
|  |  |  | Mean DFS(months) | | adjusted HR  (95%CI) | p-value | adjusted HR  (95%CI) | p-value | | Mean  OS(months) | | adjusted HR  (95%CI) | p-value | adjusted HR  (95%CI) | p-value |
| Age, years | ≤65 | 17(65) | 59.1±17.3 | | Ref | 0.822 | Ref | 0.923 | | 72.0±15.6 | | Ref | 0.183 | Ref | 0.513 |
|  | >65 | 9(35) | 55.4±16.2 | | 0.89(0.33-2.43) |  | 0.93(0.21-4.12) |  | | 155±34.2 | | 0.36(0.08-1.62) |  | 0.54(0.08-3.47) |  |
| Sex | Female | 9(35) | 73.8±22.1 | | Ref | 0.679 | Ref | 0.932 | | 129±30.6 | | Ref | 0.354 | Ref | 0.731 |
|  | Male | 17(65) | 49.4±15.1 | | 1.24(0.45-3.37) |  | 1.06(0.28-3.96) |  | | 69.1±15.1 | | 1.75(0.54-5.72) |  | 1.29(0.30-5.53) |  |
| Smoking, pys | ≤20 | 12(46) | 60.4±17.9 | | Ref | 0.893 | Ref | 0.878 | | 78.9±16.5 | | Ref | 0.990 | Ref | 0.718 |
|  | >20 | 14(54) | 57.0±18.4 | | 1.07(0.41-2.78) |  | 1.12(0.26-4.83) |  | | 108±28.5 | | 1.01(0.34-3.01) |  | 0.76(0.17-3.41) |  |
| Pathology | ADC | 15(58) | 57.7±14.6 | | Ref- | 0.901 | Ref | 0.795 | | 107±22.3 | | Ref | 0.930 | Ref | 0.826 |
|  | SQCC | 10(38) | 68.5±26.2 | | 0.94(0.32-2.71) |  | 0.81(0.16-4.06) |  | | 105±26.0 | | 0.84(0.25-3.52) |  | 1.22(0.21-6.95) |  |
| T Stage | T1 | 4(15) | 43.5±21.0 | | Ref | 0.951 | Ref | 0.764 | | 68.5±15.7 | | Ref | 0.908 | Ref | 0.879 |
|  | ≥T2 | 22(85) | 63.5±15.2 | | 1.04(0.29-3.64) |  | 1.28(0.26-6.21) |  | | 110±21.9 | | 0.83(0.25-3.38) |  | 1.13(0.25-5.14) |  |
| N stage | N0 | 1(4) | 13.0±0.0 | | Ref | 0.479 | Ref | 0.295 | | - | | SvcbvxRef | 0.778 | Ref | 0.778 |
|  | ≥N1 | 25(96) | 64.4±14.1 | | 0.47(0.06-3.75) |  | 0.26(0.02-3.22) |  | | - | | -21.5(0.0-3.83E+10) |  | 21.5(0.0-.83E+10) |  |
| Platinum-based | No | 12(46) | 80.3±22.7 | | Ref | 0.636 | Ref | 0.510 | | 136±29.1 | | Ref | 0.285 | Ref | 0.306 |
| chemotherapy | Yes | 14(54) | 47.3±13.9 | | 1.27(0.47-3.47) |  | 1.66(0.37-7.45) |  | | 64.1±14.4 | | 1.91(0.58-6.26) |  | 2.21 (0.48-10.1) |  |
| Romo1 | Low | 8(31) | 37.9 ± 16.2 | | Ref | 0.416 | Ref | 0.802 | | 57.9±17.7 | | Ref | 0.646 | Ref | 0.964 |
|  | High | 18(69) | 69.3 ± 17.0 | | 0.66(0.24-1.80) |  | 0.83(0.19-3.69) |  | | 111±22.4 | | 0.76(0.23-2.48) |  | 1.04(0.19-5.59) |  |

DFS: disease free survival; OS: overall survival, HR: hazard ratio, CI: confidence interval; pys: pack-years
