# Supplementary figures and images for: Reactive oxygen species modulator 1 expression predicts lymph node metastasis and survival in early-stage non-small cell lung cancer
Source: PLoS One. 2020 Dec 1;15(12):e0239670. doi: 10.1371/journal.pone.0239670 (PMC7707601; doi:10.1371/journal.pone.0239670)

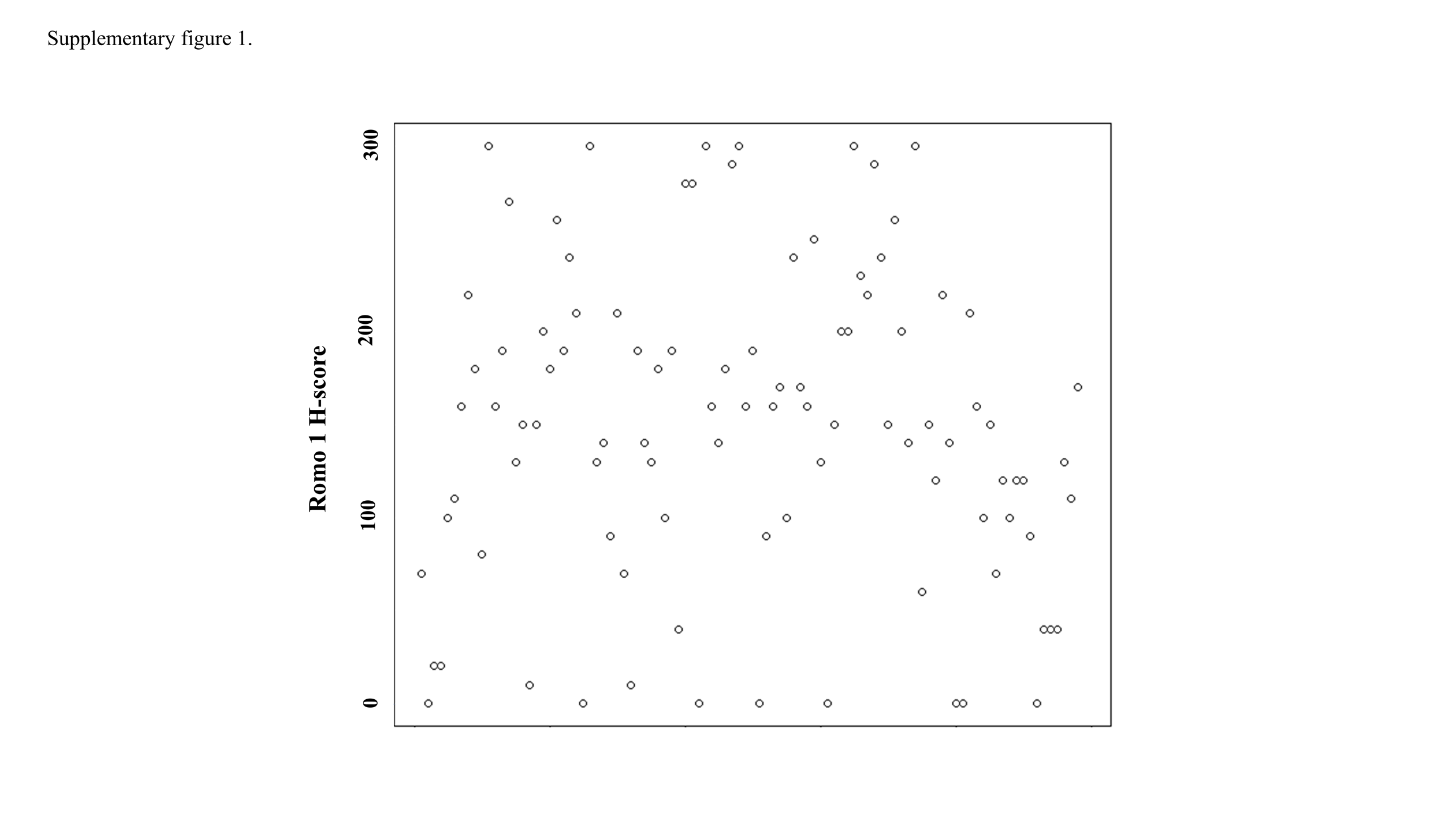

Supplement: S1 Fig — (TIF) [file pone.0239670.s003.tif]
